# Effects of Dendrimer-microRNA Nanoformulations against Glioblastoma Stem Cells

**DOI:** 10.3390/pharmaceutics15030968

**Published:** 2023-03-17

**Authors:** Nadezhda Knauer, Mariya Meschaninova, Sajjad Muhammad, Daniel Hänggi, Jean-Pierre Majoral, Ulf Dietrich Kahlert, Vladimir Kozlov, Evgeny K. Apartsin

**Affiliations:** 1 Research Institute of Fundamental and Clinical Immunology, 630099 Novosibirsk, Russia; 2 Clinic for Neurosurgery, Medical Faculty, Heinrich-Heine University Medical Center Düsseldorf, 40225 Düsseldorf, Germany; 3 Institute of Chemical Biology and Fundamental Medicine SB RAS, 630090 Novosibirsk, Russia; 4 Laboratoire de Chimie de Coordination, CNRS, 205 Route de Narbonne, CEDEX 04, 31077 Toulouse, France; 5 Molecular and Experimental Surgery, Clinic for General-, Visceral-, Vascular-, and Transplant-Surgery, Medical Faculty, University Hospital Magdeburg, 39120 Magdeburg, Germany; 6 Univ. Bordeaux, CNRS, Bordeaux INP, CBMN, UMR 5248, 33600 Pessac, France

**Keywords:** dendrimers, microRNA, nucleic acid therapeutics, 3D tumor models, glioblastoma, tumor stem cells, surface markers, nanomedicine

## Abstract

Glioblastoma is a rapidly progressing tumor quite resistant to conventional treatment. These features are currently assigned to a self-sustaining population of glioblastoma stem cells. Anti-tumor stem cell therapy calls for a new means of treatment. In particular, microRNA-based treatment is a solution, which in turn requires specific carriers for intracellular delivery of functional oligonucleotides. Herein, we report a preclinical in vitro validation of antitumor activity of nanoformulations containing antitumor microRNA miR-34a and microRNA-21 synthetic inhibitor and polycationic phosphorus and carbosilane dendrimers. The testing was carried out in a panel of glioblastoma and glioma cell lines, glioblastoma stem-like cells and induced pluripotent stem cells. We have shown dendrimer-microRNA nanoformulations to induce cell death in a controllable manner, with cytotoxic effects being more pronounced in tumor cells than in non-tumor stem cells. Furthermore, nanoformulations affected the expression of proteins responsible for interactions between the tumor and its immune microenvironment: surface markers (PD-L1, TIM3, CD47) and IL-10. Our findings evidence the potential of dendrimer-based therapeutic constructions for the anti-tumor stem cell therapy worth further investigation.

## 1. Introduction

Despite being one of the most developed clinical fields, oncology still meets challenges related to certain malignancies which are aggressive, resistant to therapeutics and have poor prognosis. Glioblastoma belongs to this category of hard-to-treat tumors [1,2] and nowadays these properties are supposed to be related to a very specific cell type—glioblastoma stem cells (GSCs), a subpopulation of tumor cells capable of self-sustaining and associated with tumor formation, progression and recurrence [3,4,5,6,7]. It is also currently suggested that tumor stem cells may play a key role in the development of glioblastoma resistance to chemotherapy [5,6,7,8,9,10]; different model tumor lines may have different sensitivity to drugs [11,12].

To meet the challenges of tumor nanomedicine, a therapeutic tool is needed to efficiently influence metabolic pathways in the target cells, while the idea of affecting several targets seems attractive. These requirements are met by microRNAs—short (about 18–22 nucleotides) endogenous non-coding RNAs, actively involved in the regulation of gene expression of cell cycle regulators, proliferation, differentiation, and metabolism [13,14,15,16]. The functional activity of microRNAs is mainly related to the suppression of translation and mRNA degradation (note that in some cases translation enhancement upon mRNA binding to miRISC is described) [13,17,18,19,20]. At the same time, other ways of microRNA regulatory activity in the cell have also been shown, e.g., sequestration of mRNA in the regions of the cell where no active protein synthesis occurs, ribosome removal from mRNA and interruption of protein synthesis, as well as protease involvement [15,19,20].

Thus, microRNAs can be considered as multifunctional and flexible modulators for regulating the activity of immunocompetent or tumor cells, as well as their interaction. These characteristics make microRNAs a promising tool for researchers trying to influence metabolic pathways in cells during the formation of pathological conditions. However, therapeutic constructs based on nucleic acids face several challenges, such as low cellular uptake and risk of rapid degradation in biological media due to the activity of nucleases [19,21,22]. This can be partially solved by performing additional chemical modifications [22,23] or by using a nanocarrier for efficient oligonucleotide protection and enhanced uptake. The search for new oligonucleotide delivery vehicles that would efficiently deliver therapeutic components to target tissues and cells while possessing a high biocompatibility profile is still emerging. At the same time, their production should be relatively simple and scalable for subsequent commercial application. For instance, dendrimers, a class of polymeric molecules, meet these requirements.

Dendrimers are the common name for a large family of highly symmetric hyperbranched molecules of various chemical origins [24]. They consist of a central core and multiple “branches” originating from the center and having several bifurcation points; the number of bifurcation points is the same on all “branches” and determines the dendrimer generation [24,25]. The characteristics of dendrimers and peculiarities of their production allow the necessary functional groups to be introduced into the structure, achieving the specified chemical properties [14,26]. Compared to other polymeric carriers, the synthesis of dendrimers is well controlled at each step and can be performed in large volumes, and the resulting reaction products are monodisperse—have a given size and molecular weight, which is extremely important in the commercial production of substances for real therapeutic practice, either as drugs themselves, or as carriers for therapeutic entities or as drug depots for sustained release [27,28,29,30].

For nucleic acid transport, cationic dendrimers are of great interest: cationic functional groups on the surface allow noncovalent binding of nucleic acid fragments, to form dendriplexes, whose characteristics may depend on the charge ratio in the dendrimer/nucleic acid system [25,27,31,32,33,34,35]. On the other hand, it should be noted that these molecules have their own toxic effect already in medium concentrations [27,36]. It is assumed that cytotoxicity depends not only on the chemical origin of the molecule, but also on the generation and molecular weight, which is characteristic of all such polymers [14]. However, high generation dendrimers usually have greater permeation activity [31], so a balance between biocompatibility and transport efficiency must be found.

In the current study we investigated the biological effects of cationic phosphorus and carbosilane dendrimers and their complexes with therapeutic oligonucleotides—miR-34 and miR-21 synthetic inhibitor—on several types of GSCs in comparison to a “standard” glioblastoma cell (U87 culture) and non-tumor cells (human induced pluripotent stem cells, iPSCs). We assessed the effects of molecules and their complexes on cells’ viability, parameters of apoptosis induction, IL-10 secretion and expression of surface molecules, characterizing interactions of tumor cells with the immune microenvironment (PD-L1, TIM-3, CD47).

## 2. Materials and Methods

### 2.1. Cell Lines

In the current study the following cell lines were used: human glioblastoma stem like cell lines BTSC233, JHH520, NCH644, GBM1; human glioblastoma cell line U87; human induced pluripotent stem cells iPSCs. Cells were kindly provided by colleagues: BTSC233 (M.S. Carro, Freiburg University, Freiburg im Breisgau, Germany); JHH520 (G. Riggins, Johns Hopkins, Baltimore, MD, USA); NCH644 (C. Herold-Mende, Heidelberg University, Heidelberg, Germany); GBM1 (A. Vescovi, San Raffaele Hospital, Milano, Italy); U87 (A. Weyerbrock, Department General Neurosurgery, Medical Center Freiburg, Germany). Ethical approval was granted by the ethics commission of the Medical Faculty of the Heinrich-Heine University (study ID 5841R).

Tumor cells were cultivated as neurospheres in accordance with previously described protocols [37,38] in standard conditions (humidified 37 °C, 5% CO_2_) in a DMEM media (high-glucose, no pyruvate, Thermo Fisher, Waltham, MA, USA) with F12 (3:1) and 1X B27 supplements (both Thermo Fisher, Waltham, MA, USA), 20 ng/mL human EGF, 20 ng/mL human VGF (both Peprotech, Hamburg, Germany), 5 µg/mL heparin (Sigma- Aldrich, Taufkirchen, Germany), 1X penicillin/streptomycine (Sigma-Aldrich, Taufkirchen, Germany).

iPS cells were cultivated in a serum-free mTeSR medium (Stemcell Technologies, Saint Égrève, France) without antibiotics in flat-bottomed culture plates covered by vitronectin (Gibco, New York, NY, USA).

For all assays, a volume-adjusted medium was used as NTC treatment.

### 2.2. Therapeutic Formulations

**Figure 1:**
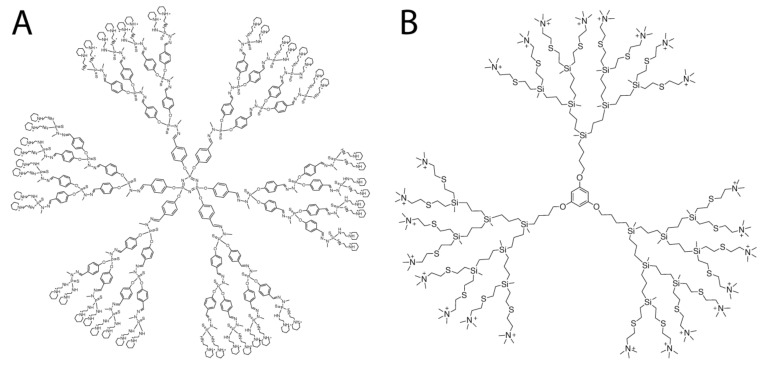
Molecular structures of cationic phosphorus dendrimer (**A**) and carbosilane dendrimer (**B**) used in the study.

The 3rd generation cationic dendrimers were used: phosphorus dendrimer AE2G3 (it was synthetized as previously described in [25,39]) and carbosilane dendrimer BDEF33 (it was synthetized as previously described in [40]) (see Figure 1). As therapeutic oligonucleotides miR-34a (5′-r(UGGCAGUGUCUUAGCUGGUUGU), r—ribonucleotides) and synthetic inhibitor of miR-21 (amiR-21, 5′-m(UCAACAUCAGUCUGAUAAGCUA), m—(2′-O-methylribo)nucleotides) were chosen.

The dendrimers were dissolved in ultrapure water (milli-Q) to a final concentration of 1 mM and stored at +4 °C. Oligonucleotides were dissolved in ultrapure water (milli-Q) to a final concentration of 20 mM and stored at −20 °C. Temozolomide (TMZ, Sigma- Aldrich, Taufkirchen, Germany) was dissolved in DMSO (dimethyl sulfoxide, Sigma-Aldrich, Taufkirchen, Germany) to a final concentration of 20 mM and stored at −20 °C.

To prepare the dendriplex solutions, the oligonucleotide solutions were mixed according to the optimal charge ratios found previously [25,41], 10-fold cation excess was used; the RNA concentrations were 25 nM, 50 nM, 100 nM, and 150 nM. The complexes were incubated in the dark for 15 min at room temperature and used for subsequent work (dendriplexes were prepared anew for each experiment). Physicochemical characteristics of complexes obtained (hydrodynamic diameter 35–45 nm; PDI 0.21–0.25; zeta potential +14 mV for AE2G3-miR complexes, +1.5 mV for BDEF33-miR complexes) were in accordance with those observed earlier [41].

Lipofectamine 3000 (Lipo, Invitrogen, Waltham, MA, USA) was used as a control nucleic acid carrier; nucleic acid complexes were prepared according to the manufacturer’s recommendations.

### 2.3. MTT-Assay

Cells were cultured in 96-well flat-bottomed culture plates at a ratio of 10,000 cells in 100 μL of complete medium (tumor cells), 100,000 cells in 1000 μL of complete medium (iPSCs). The cells were incubated for 72 h in the presence of free dendrimers or their complexes under standard conditions (T = 37 °C, 5% CO_2_, humidified atmosphere). Untreated cells (NTC, non-treated control) were used as a control sample, medium was used as a blank control. Each point had 3–5 technical repeats.

After incubation, 10 μL of MTT solution (thiazolyl blue tetrazolium bromide, Taufkirchen, Germany) in DMSO (0.5 mg/mL) was added to the wells, carefully pipetted, and incubated for 3 h in a CO_2_ incubator under standard conditions (T = 37 °C, 5% CO_2_, humidified atmosphere). A sample with an identical volume of complete medium was used as a background control. Then 100 μL MTT Lysis buffer containing isopropanol (VWR, Langenfeld, Germany), Triton X (Sigma Aldrich, Taufkirchen, Germany) and HCl (Roth, Karlsruhe, Germany) was added to the wells (50 mL buffer: 45 mL 99% isopropanol, 5 mL Triton X-100, 330 μL 25% hydrochloric acid), carefully pipetted, incubated at room temperature in the dark with constant stirring for 20 min. After that, the optical absorbance values were detected on a plate reader (Paradigm plate reader, Molecular Devices, San Jose, CA, USA) at 570 nm and 650 nm according to the manufacturer’s recommendations. On the graphs, data are presented as a percentage of value measured for the NTC.

### 2.4. Apoptosis Assay

Muse^®^ Annexin V & Dead Cell Kit (Luminex, Austin, TX, USA) were used to work with tumor cells and iPSCs, followed by analysis with Guava^®^ Muse^®^ Cell Analyzer (Luminex, Austin, TX, USA). We analyzed apoptosis induction parameters as the ratio of live (AnV−/PI−), early apoptotic (AnV+/PI−) and late apoptotic/necrotic (AnV+/PI+) cell populations.

Cells at 5 × 10^5^/mL were incubated for 72 h in a 12-well plate in the presence of free dendrimers (10 μM) or dendriplexes (150 nM RNA equivalent according to charge ratio; or individual components in appropriate concentrations) under standard conditions (T = 37 °C, 5% CO_2_, humidified atmosphere). After culturing, cells were harvested and washed in a standard manner.

Then, 100 μL of Muse Annexin V & Dead Cell Reagent (Luminex, Austin, TX, USA) were added to a cell suspension containing 1–5 × 10^5^ in 100 μL PBS (Thermo Fisher Scientific, Waltham, MA, USA), thoroughly mixed, and incubated for 20 min at room temperature in the dark. Then samples were analyzed.

### 2.5. Internalization Assay

Cells were cultured for 4 h in 12-well flat-bottomed plates (100,000 cells per 500 μL of medium) in the presence of free dendrimers, fluorescently labeled amiR-155-FAM microRNA (150 nM) and their complexes (150 nM RNA equivalent according to the charge ratio) under standard conditions (T = 37 °C, 5% CO_2_, humidified atmosphere). The cells were then harvested, washed twice with cold PBS (Thermo Fisher Scientific, Waltham, MA, USA) containing 10% FCS (Thermo Fisher Scientific, Waltham, MA, USA) and 0.02% EDTA (Sigma Aldrich) (centrifuged at 1200 rpm for 5 min). To remove molecules adhering to the cell surface, cells were treated with acidic glycine (Sigma Aldrich) buffer for 30 s (50 mmol/L, pH 3.0) between washes. The cells were fixed with 4% PFA (Sigma Aldrich) solution and analyzed. Cytometric analysis was performed on a CyAn Daco flow cytometer (Daco, Beckman Coulter, Denver, CO, USA) machine using Summit (Beckman Coulter, Denver, CO, USA) software. Experiments were performed using three independent biological repetitions before statistical analysis.

### 2.6. Expression of Surface Markers

Phenotyping of the cells as well as evaluation of the expression of functional molecules was performed by multicolor flow cytometry on CyAn Daco, Beckman Coulter cytofluorimeters with subsequent analysis (Summit 5.2., Kaluza 2.2., Beckman Coulter software). Negative controls and FMO controls (Fluorescence-Minus-One) were used to determine the positive population when evaluating the expression of the molecules under study. Biological controls were non-treated cells (NTC, non-treated control). Tumor cell samples and iPSCs were examined in three biological replicates. The following monoclonal fluorescent-labelled antibodies were used: TIM-3-FITC (Biolegend, Amsterdam, The Netherlands), PD-L1-PE (Biolegend, Amsterdam, The Netherlands), CD47-APC (BD, Eysins, Switzerland) and Annexin V-Pacific Blue antibodies (Biolegend, Amsterdam, The Netherlands) for distinguishing dead and live cells.

The studied cells (GBM1, NCH644, U87, iPSCs) were cultured for 72 h in the presence of free dendrimers (1 μM) or their complexes (150 nM RNA equivalent) under standard conditions (T = 37 °C, 5% CO_2_, humidified atmosphere) in flat-bottomed culture plates.

For staining the surface molecules of the cells under study with fluorescence-labeled monoclonal antibodies, 0.3–0.5 × 10^6^ cells were added to cytometry test tubes in a volume not exceeding 100 μL. An appropriate mixture of antibodies was added to each test tube (the volume of individual antibodies was determined according to the manufacturer’s instructions) according to the experiment scheme and the controls used. The cell suspension was gently mixed on a Vortex mixer at medium speed, incubated in the dark at room temperature for 20 min. The excess of antibodies was washed by adding 2 mL of PBS buffer with 0.02% EDTA and 1% FCS (centrifuged at 1200 rpm for 5 min), and the supernatant was removed. The cells were resuspended in 300–350 μL of buffer and used for further analysis.

### 2.7. IL-10 Secretion

The evaluation of IL-10 secretion by tumor cells (NCH644) was performed with the Human IL-10 ELISA MAX Deluxe kit (Biolegend, Amsterdam, The Netherlands). After cells were cultured with the molecules under study and their complexes (see protocol above), supernatants were taken and the cytokine level was analyzed according to the manufacturer’s recommendations. The measured optical absorbance values were expressed as a percentage of the values obtained for the untreated control.

### 2.8. Data Analysis and Visualization

Statistical analysis of the data was performed using Statistica 8.0 (StatSoft) and GraphPad Prism 7 (GraphPad Software) with nonparametric statistical methods. A Mann–Whitney test was used to assess the significance of differences between independent groups. Differences were considered significant at a significance level *p* < 0.05. Data were visualized using GraphPad Prism 7 software (GraphPad Software).

## 3. Results

### 3.1. Effects of Dendrimers on Cell Viability

**Figure 2:**
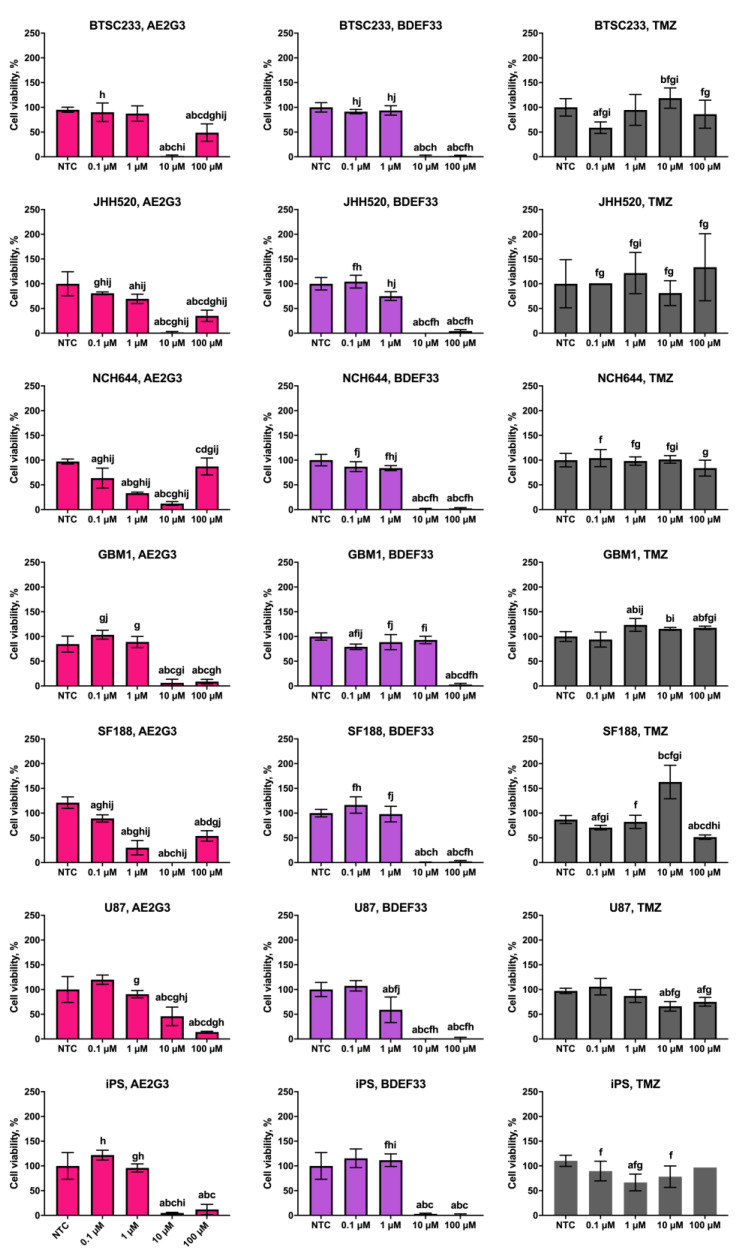
Assessment of effects of cationic phosphorus (AE2G3) and carbosilane (BDEF33) dendrimers on the viability of glioblastoma tumor cell lines and iPS cells (iPSCs) after 72 h co-culture compared with the standard chemotherapy drug temozolomide (TMZ), data are presented as a percentage of value for the NTC. Letters mark significant differences (*p* < 0.05): “a” compared with non-treated control (NTC); “b” compared with dendrimer at 0.1 μM concentration; “c” compared with dendrimer at 1 μM concentration; “d” compared with dendrimer at 10 μM concentration; “f” compared with free AE2G3; “g” compared with free BDEF33; “h” compared with free temozolomide; “i” compared with U87; “j” compared with iPSCs.

**Figure 3:**
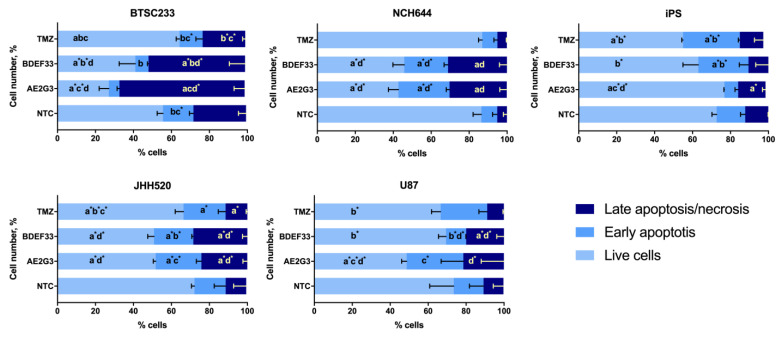
Assessment of apoptosis induction parameters of glioblastoma tumor cell lines and iPSCs after 72 h of co-culture with free dendrimers compared to standard chemodrug temozolomide (TMZ); concentration of substances tested is 10 μM. NTC—non-treated control, untreated cells. Letters mark significant differences (*p* < 0.05), mark * denotes differences at *p* = 0.05: “a” compared with non-treated control (NTC); “b” compared with free AE2G3; “c” compared with free BDEF33; “d” compared with free temozolomide.

Cell lines under study demonstrated dose-dependent sensitivity to dendrimers, with cytotoxic activity of AE2G3 slightly exceeding that of BDEF33 (see Figure 2 and Supplementary Materials). It is worth noting that temozolomide has lower activity than dendrimers, and tumor stem cell cultures are more sensitive than the “standard” U87 line, which may be interesting for the tasks of anti-tumor therapy in the future. These data are consistent with the results obtained in the study of apoptosis (see Figure 3).

It is worth noting that even in a test such as MTT we observe differences in the ability of different tumor cell types to uptake dendrimer-based constructions. Apparently, in a serum-free medium, AE2G3 in high concentrations still forms supramolecular associations, however, they are presumably smaller in size. U87 and GBM1 cells responded with decreased viability to the use of AE2G3 dendrimer at 100 μM concentration, suggesting that the formed particles are effectively internalized by cells and exert cytotoxic effects. At the same time, cells of BTSC233, JHH520, and NCH644 lines presumably uptake these associates less efficiently—therefore, we observed an increase in cell viability.

With respect to glioblastoma lines, several interesting patterns were revealed. It was shown that the studied cell lines showed greater sensitivity to the investigated dendrimers than to temozolomide, the classical drug used in glioblastoma chemotherapy. This effect is already evident at medium concentrations (10 µM). In addition, cultures of glioblastoma tumor stem cells appeared to be more sensitive to the studied dendrimers compared to U87 cells, which is of particular interest in light of the information on the role of tumor stem cells in providing tumor resistance to therapy and its metastasis.

Several cultures (BTSC233, JHH520, NCH644) showed a paradoxical increase in viability when using high concentrations of AE2G3 (100 μM), while the viability of GBM1, U87 was characterized by the absence of such peak. We hypothesize that supramolecular associations of AE2G3 formed under high concentration conditions are internalized differently by different tumor cell types.

When studying the parameters of tumor cell apoptosis induction after culturing with free dendrimers, we observed a decrease in the number of living cells, and an increase in subpopulations of early-apoptotic and late-apoptotic/necrotic cells compared to controls (*p* = 0.05) (see Figure 3 and Supplementary Materials). At the same time, both dendrimers under study exhibited comparable activity. These experiments generally reproduced the effect observed in previous cell viability studies—dendrimers have a higher toxic effect than temozolomide. The studied dendrimers showed either similar activity against tumor cells or AE2G3 had a slightly higher toxic effect.

### 3.2. Effect of Dendrimers on the Expression of Tumor Cell Surface Markers

The PD-L1, ligand of the PD-1 receptor of immunocompetent cells, is associated with suppression of the anti-tumor immune response [42,43]. Higher PD-L1 expression correlates with disease severity and can be used as a marker of poor prognosis in gliomas [43,44,45,46,47]. Effects on the PD-1/PD-L1 axis and blocking these interactions have shown good results, reducing the number of depleted T cells in the microenvironment (which improves cytotoxic anti-tumor effects) and promoting immunological memory [48], so inhibitory therapy targeting PD-1 and PD-L1 plays an important role in current glioblastoma treatment protocols [42,49,50]. However, as data have accumulated, the question of how the expression of checkpoint molecules (such as PD-L1) changes with chemotherapy and how this might affect treatment efficacy has increasingly come up. On the one hand, Heynckes and colleagues found a decrease in PD-L1 expression in glioblastoma samples after temozolomide treatment, blocking JAK-STAT signaling potentially associated with worse prognosis [42]. On the other hand, Wang et al., observed a more active departure of glioblastoma cells from immune surveillance after temozolomide therapy by increasing PD-L1 expression [51]. Moreover, Karachi group experiments demonstrated that the use of temozolomide at standard therapeutic doses in a mouse glioblastoma model resulted in increased expression of PD-L1 on CD4+, CD8+ T cells and T regulatory cells, as well as increased expression of other cellular depletion markers LAG-3 and TIM-3 [49]. A possible explanation for this contradiction could be that PD-L1 could potentially prove to be a marker of cellular stress following the use of chemodrug formulations [52]. It has been suggested that the combination of temozolomide and checkpoint molecule inhibitors may help improve patient outcomes [51].

TIM-3 (T cell immunoglobulin domain and mucin domain 3) is a negative checkpoint-regulator with functions similar to PD-L1; its expression was detected on type 1 CD4+ T-helper cells and cytotoxic CD8+ T-cells; later TIM3 expression was described on tumor cells (leukemia, glioblastoma) as a marker of worsening disease prognosis and resistance to chemotherapy [53,54,55]. It was hypothesized that TIM3 can be considered as one of the markers of tumor stem cells in glioblastoma [53]. TIM3 signaling is associated with differentiation of myeloid suppressor cells into tumor-associated macrophages and development of “tumor niche” [56]. TIM3 expression is associated with glioblastoma cells resistance to temozolomide therapy, moreover, its use leads to an increase in TIM3 expression; at the same time, suppression of TIM3 expression is associated with higher level of apoptosis [55].

CD47 molecule binds to SIRPα on macrophages, neutrophils and dendritic cells, leading to the suppression of their phagocytic and antigen-presenting activities, thus acting as a “do not eat me” signal [57]. High expression of CD47 on tumor stem cells of leukemia and glioblastoma has been described [58,59]. In general, increased CD47 expression on tumor cells is associated with a worse prognosis in glioblastoma [58].

**Figure 4:**
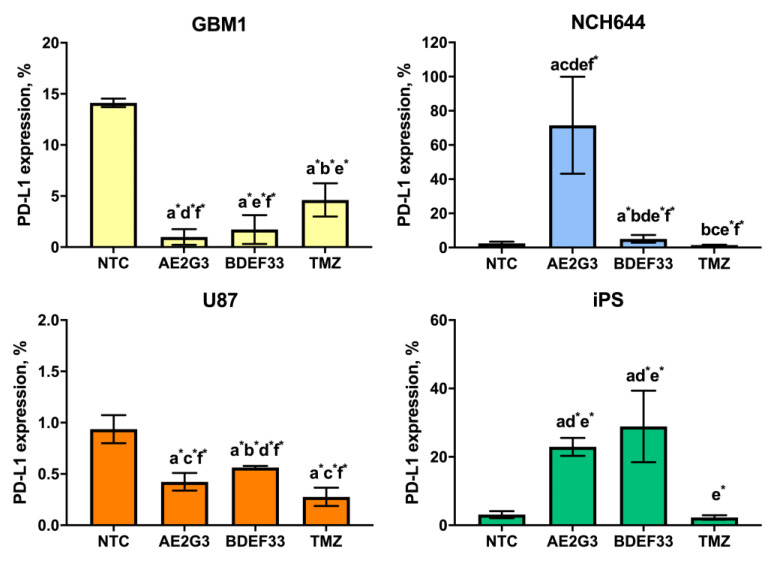
Assessment of PD-L1 marker expression on the surface of glioblastoma tumor cell lines and iPSCs after 72 h of co-culture with free dendrimers compared to standard chemodrug temozolomide (TMZ); concentration of the substances tested is 3 μM. NTC—non-treated control, untreated cells. Letters mark significant differences (*p* < 0.05), mark * denotes differences at *p* = 0.05: “a” compared with non-treated control (NTC); “b” compared with free AE2G3; “c” compared with free BDEF33; “d” compared with free temozolomide; “e” compared with U87; “f” compared with iPS.

When studying PD-L1 expression in cultures of glioblastoma lines, the results differed depending on the cell line (see Figure 4 and Supplementary Materials). Thus, on GBM1 cells use of dendrimers reduced PD-L1 expression (*p* = 0.05), while the addition of AE2G3 had a higher effect. At the same time, PD-L1 expression on NCH644 cells increased when cultured with AE2G3 (*p* = 0.01), BDEF33 and TMZ did not show such a pronounced effect; remarkably, these results are also consistent with the data described above on the higher sensitivity of NCH644 cells to AE2G3.

**Figure 5:**
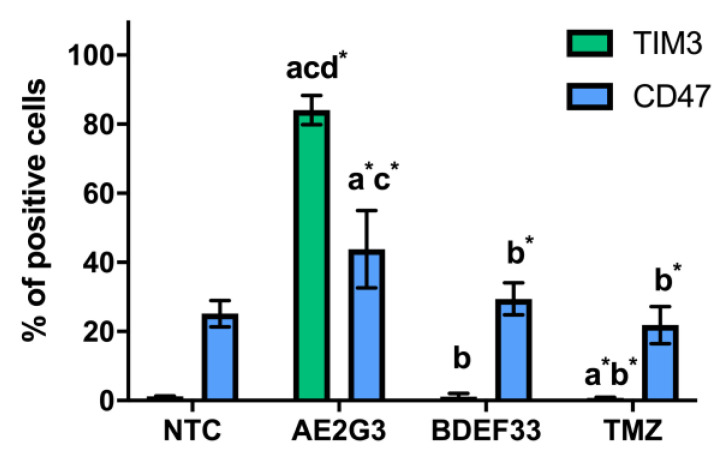
Evaluation of TIM3, CD47 expression on the surface of NCH644 tumor cell lines after 72 h of co-culture with free dendrimers compared to standard chemodrug temozolomide (TMZ); concentration of substances tested is 3 μM. NTC—non-treated control, untreated cells. Letters mark significant differences (*p* < 0.05), mark * denotes differences at *p* = 0.05: “a” compared with non-treated control (NTC); “b” compared with free AE2G3; “c” compared with free BDEF33; “d” compared with free temozolomide.

PD-L1 expression on iPSCs increased with AE2G3 (*p* = 0.005) and BDEF33 (*p* = 0.03). Note that the expression of TIM3 and CD47 markers on NCH644 tumor cells also increased after culturing with dendrimers (see Figure 5 and Supplementary Materials): NCH644 cells responded to the use of AE2G3 by increased TIM3 expression (*p* = 0.03), NCH644 cells responded predominantly to AE2G3 addition, as in the case of PD-L1.

### 3.3. Effect of Dendrimers on IL-10 Secretion by Tumor Cells

**Figure 6:**
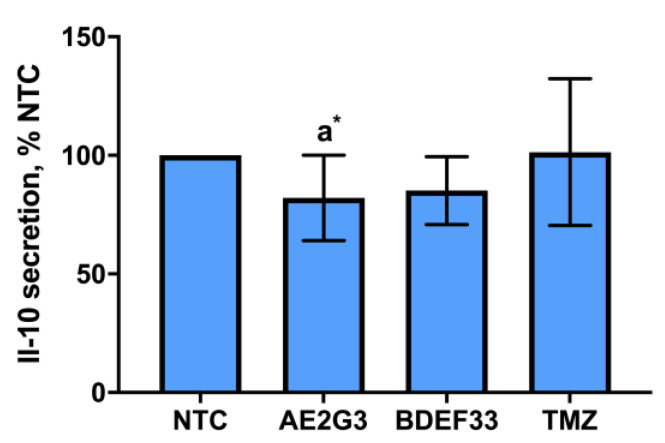
Assessment of IL-10 cytokine secretion in NCH644 tumor cell cultures after 72 h of co-culture with free dendrimers compared to standard chemodrug temozolomide (TMZ); NTC—non-treated control, untreated cells. Letters mark significant differences (*p* < 0.05), mark * denotes differences at *p* = 0.05: “a” compared with non-treated control (NTC).

An important parameter describing the relationship between the tumor and its microenvironment (in particular, the immune microenvironment) is the production of IL-10, a cytokine with an immunosuppressive effect. To assess the effects of dendrimers on IL-10 production, we have chosen NCH644 line as a representative example of glioblastoma stem-like cells. We found a slight decrease in IL-10 production by NCH644 cells when cultured with AE2G3 (*p* = 0.05), but not BDEF33 and TMZ molecules (see Figure 6 and Supplementary Materials).

### 3.4. Evaluation of Dendrimer-microRNA Complexes Internalization in Tumor Cells

**Figure 7:**
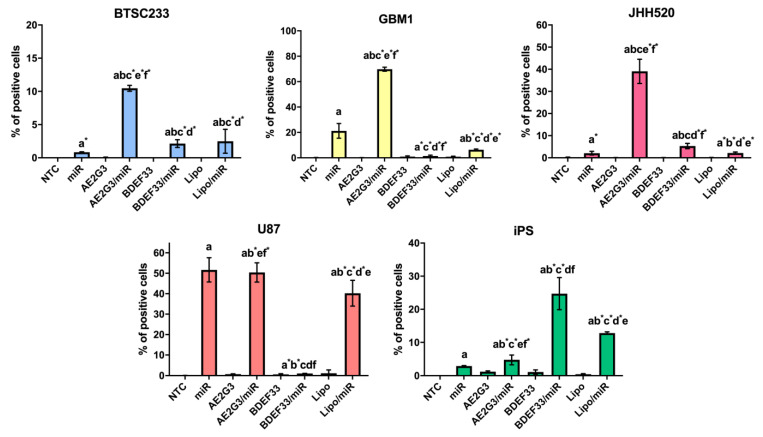
Assessment of the efficiency of internalization of dendrimer complexes with fluorescently labeled microRNA (miR-155-FAM) into tumor cells and iPS cells compared to Lipofectamine 3000 (Lipo) (4 h). Letters mark significant differences (*p* < 0.05), mark * denotes differences at *p* = 0.05: “a” compared with non-treated control (NTC)); “b” compared with free dendrimer; “c” compared with free miR; “d” compared with AE2G3/miR; “e” compared with BDEF33/miR; “f” compared with Lipo/miR.

Cultures representing glioblastoma tumor stem cell models internalize microRNA complexes with AE2G3 most actively, with internalization efficiency exceeding that of both free microRNA and complexes with dendrimer BDEF33 and lipofectamine 3000, which is standard for transfection (see Figure 7 and Supplementary Materials). BDEF33 also has relatively low activity for microRNA delivery to U87 tumor cells, while the cells efficiently internalize both free microRNA and microRNA complexes with AE2G3 and lipofectamine 3000. At the same time, it was BDEF33 that proved to be the most effective microRNA carrier for delivery into iPSCs (this may also explain the greater toxicity of BDEF33 for this cell type shown earlier).

Thus, we demonstrated that dendrimers are efficient microRNA carriers. Similar results were also previously observed for the delivery of siRNA into glioblastoma stem cells, HeLa cells, and induced pluripotent stem cells, and the transport activity of dendrimers was higher than that of lipofectamine 2000 and lipofectamine 3000 [27,38].

### 3.5. Evaluation of the Cytotoxic Effect of Dendriplexes on Tumor Cells

Given the antitumor nature of the activity of the oligonucleotides under study and the dose-dependent toxic activity of dendrimers against tumor cells demonstrated earlier, the most expected observation would have been a dose-dependent decrease in cell viability of the cultures studied and the induction of apoptosis. However, the actual picture was somewhat different from that expected.

**Figure 8:**
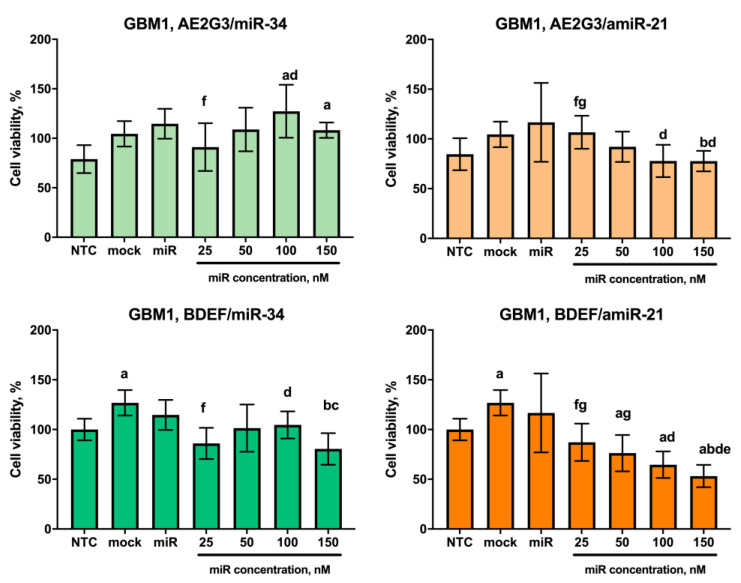
Evaluation of the effects on the viability of GBM1 tumor cell line after 72 h co-culture with dendriplexes, data are presented as a percentage of value for the NTC. Letters mark significant differences (*p* < 0.05): “a”, compared with non-treated control (NTC); “b”, compared with free carrier; “c”, compared with free miR; “d”, compared with dendriplex containing 25 nM RNA; “e” compared to a dendriplex containing 50 nM RNA; “f” compared to a dendriplex containing 100 nM RNA; “g” compared to a dendriplex containing 150 nM RNA.

With respect to the GBM1 line cells, BDEF33 complexes with miR-34 and amiR-21 were only slightly more toxic than AE2G3 complexes (see Figure 8 and Supplementary Materials). At the same time, we observed a dose-dependent decrease in cell viability with complexes containing amiR-21; this effect was more pronounced for the BDEF33 dendrimer, which suggests a greater sensitivity of tumor cells to this molecule.

**Figure 9:**
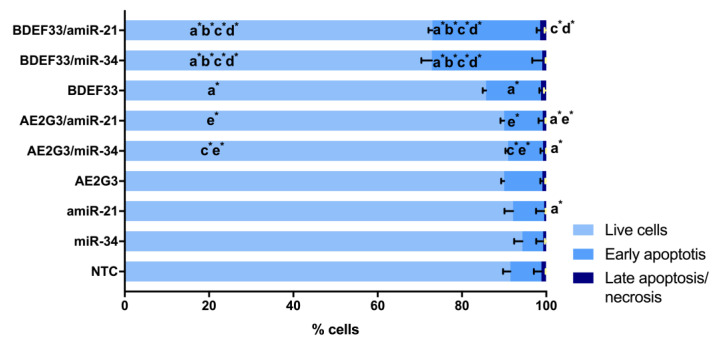
Evaluation of apoptosis induction parameters of GBM1 tumor cells after 72 h of co-culture with dendriplexes. NTC—non-treated control, untreated cells. Letters mark significant differences (*p* < 0.05), mark * denotes differences at *p* = 0.05: “a” compared with non-treated control (NTC); “b” compared with free dendrimer; “c” compared with free miR; “d” compared with AE2G3/miR complex; “e” compared with BDEF33/miR complex.

This assumption is confirmed by the data of the analysis of apoptosis parameters of the cells of this line as a tendency (*p* = 0.05): AE2G3 and its complexes showed no significant effect, whereas BDEF33 and its complexes decreased the number of living cells and increased the number of early-apoptotic cells (see Figure 9 and Supplementary Materials).

**Figure 10:**
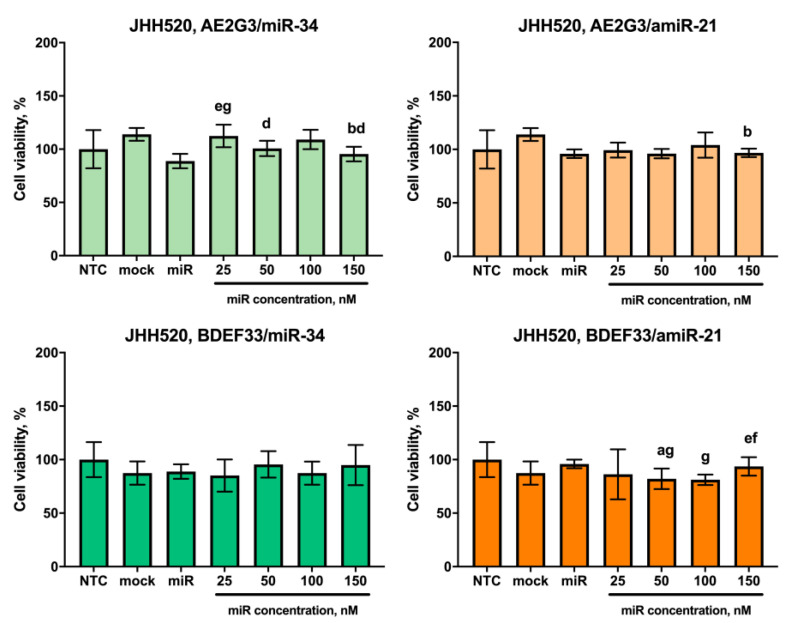
Evaluation of the effects on the viability of JHH520 tumor cell line after 72 h co-culture with dendriplexes, data are presented as a percentage of value for the NTC. Letters mark significant differences (*p* < 0.05): “a”, compared with non-treated control (NTC); “b”, compared with free carrier; “d”, compared with dendriplex containing 25 nM RNA; “e” compared to a dendriplex containing 50 nM RNA; “f” compared to a dendriplex containing 100 nM RNA; “g” compared to a dendriplex containing 150 nM RNA.

**Figure 11:**
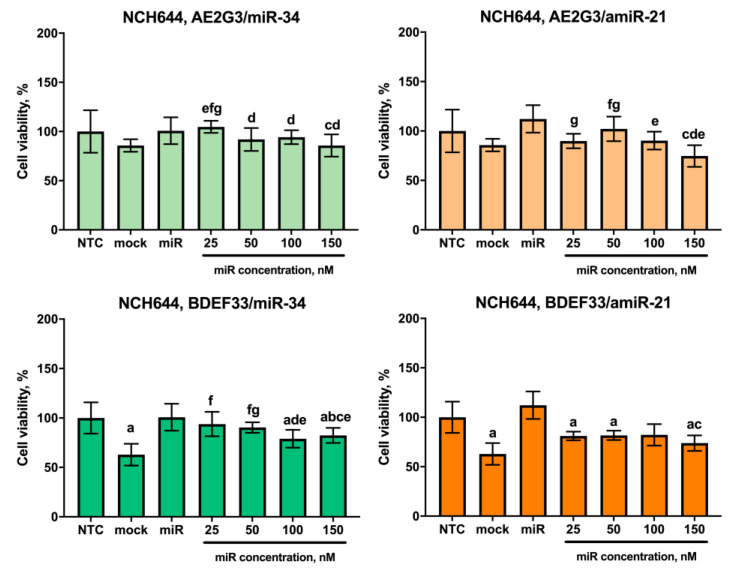
Evaluation of the effects on the viability of NCH644 tumor cell line after 72 h co-culture with dendriplexes, data are presented as a percentage of value for the NTC. Letters mark significant differences (*p* < 0.05): “a”, compared with non-treated control (NTC); “b”, compared with free carrier; “c”, compared with free miR; “d”, compared with dendriplex containing 25 nM RNA; “e” compared to a dendriplex containing 50 nM RNA; “f” compared to a dendriplex containing 100 nM RNA; “g” compared to a dendriplex containing 150 nM RNA.

Other representative cultures of the glioblastoma tumor stem cell category JHH520, NCH644 showed no significant dose-dependent sensitivity to the complexes tested, although NCH644 cells were sensitive to BDEF33 mock control (see Figure 10 and Figure 11 and Supplementary Materials). Thus, these cultures were excluded from the further experiments. Interestingly, NCH644 cells showed the same sensitivity to different dosages of dendriplexes and the effect did not change significantly when different types of RNA were used, which confirms the assumption we put forward above: the efficiency of the dendriplex is primarily determined by the properties of the dendrimer.

**Figure 12:**
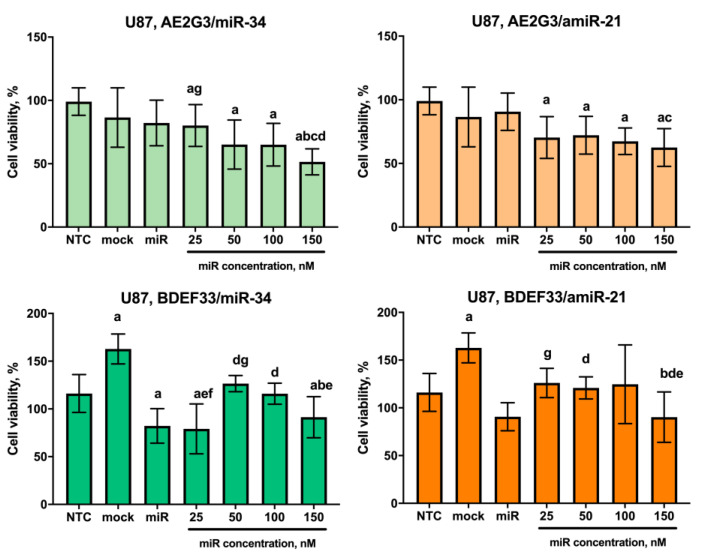
Evaluation of the effects on the viability of U87 tumor cell line after 72 h co-culture with dendriplexes, data are presented as a percentage of value for the NTC. Letters mark significant differences (*p* < 0.05): “a”, compared with non-treated control (NTC); “b”, compared with free carrier; “c”, compared with free miR; “d”, compared with dendriplex containing 25 nM RNA; “e” compared to a dendriplex containing 50 nM RNA; “f” compared to a dendriplex containing 100 nM RNA; “g” compared to a dendriplex containing 150 nM RNA.

A different picture was observed for cells of the U87 line. Here, AE2G3 complexes demonstrated a moderate cytotoxic effect, while the profiles for dendriplexes containing miR-34 and amiR-21 were similar (see Figure 12 and Supplementary Materials).

**Figure 13:**
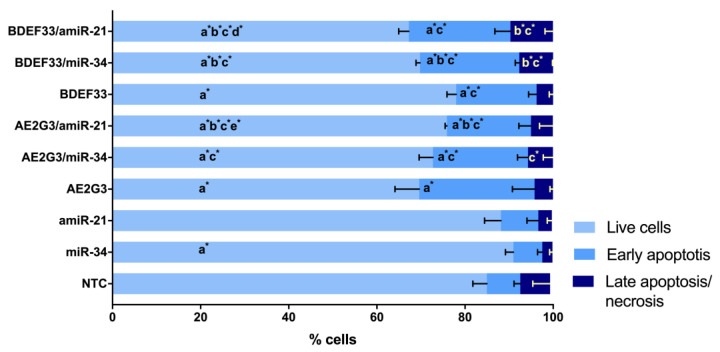
Evaluation of apoptosis induction parameters of U87 tumor cells after 72 h of co-culture with dendriplexes. NTC—non-treated control, untreated cells. Letters mark significant differences (*p* < 0.05), mark * denotes differences at *p* = 0.05: “a” compared with non-treated control (NTC); “b” compared with free dendrimer; “c” compared with free miR; “d” compared with AE2G3/miR complex; “e” compared with BDEF33/miR complex.

Analyzing apoptosis induction parameters, we observed a slight decrease in the number of living cells and an increase in the number of early apoptotic cells with both dendrimer and their complexes (*p* = 0.05) (see Figure 13 and Supplementary Materials).

### 3.6. Evaluation of the Effect of Dendriplexes on the Expression of Surface Markers of Tumor Cells

**Figure 14:**
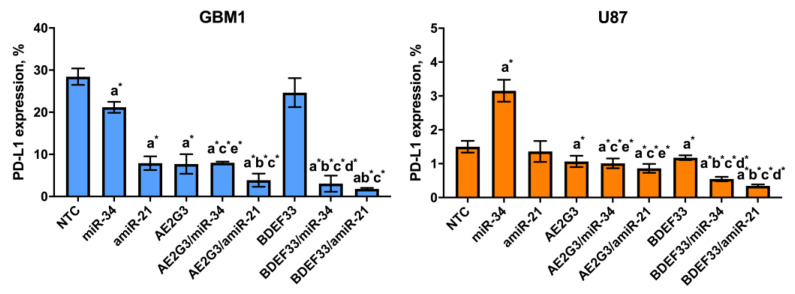
Evaluation of PD-L1 marker expression on the surface of GBM1 and U87 tumor cells after 72 h of co-culture with dendriplexes. NTC—non-treated control, untreated cells. Letters mark significant differences (*p* < 0.05), mark * denotes differences at *p* = 0.05: “a” compared with non-treated control (NTC); “b” compared with free dendrimer; “c” compared with free miR; “d” compared with AE2G3/miR complex; “e” compared with BDEF33/miR complex.

For this experiment GBM1 was chosen as a culture demonstrated decreasing of PD-L1 expression after the free dendrimer treatment; U87 was chosen as a “standard” glioblastoma cell culture. Two other cell lines under study (JHH520, NCH644) also did not demonstrate significant sensitivity to dendriplexes treatment. When evaluating PD-L1 expression on tumor cells after 72 h of cultivation with dendriplexes, we did not detect significant changes in this index on the U87 cell (see Figure 14 and Supplementary Materials). At the same time, we observed a slight decrease in PD-L1 expression on GBM1 line cells both after using free RNAs (*p* = 0.05) and after using dendrimers and their complexes (*p* = 0.05 for AE2G3 and its complexes; *p* = 0.05 for BDEF33 and BDEF33/miR-34; *p* = 0.04 BDEF33/amiR-21 compared with NTC). This correlates with the results of the dendriplex cytotoxicity assessment described above.

## 4. Discussion

Glioblastoma is known to be a rapidly progressing tumor quite resistant to conventional treatment. These features are currently assigned to glioblastoma cancer stem cells, a self-sustaining cell subset involved into tumor initiation, progression and relapse [3,4,5,6,7]. GSCs are commonly admitted being particularly responsible for the low sensitivity of glioblastoma towards chemotherapy, in particular, using temozolomide, the only chemodrug accepted in the Western world as standard of glioblastoma care [5,6,7,8,9,10]. Recent advances in the field suggest the emergence of new molecular-targeted approaches for tackling GSC. In particular, microRNA-mediated treatment seems highly promising.

microRNAs are known to be involved both into tumor development and antitumor response. In particular, miR-21 is a well-known example, its anti-apoptotic activity was first shown in the model of glioblastoma [60], and further studies confirmed its involvement in carcinogenesis of many tumors [61]. The target of miR-21 are tumor suppressor genes: TPM1 (tropomyosin 1), PDCD4 (programmed cell death 4), SERPINB5 (maspin) in breast cancer and lymphoma models; PTEN in hepatocellular carcinoma and NK/T-cell lymphoma models; TIMP3 (tissue inhibitor of metalloproteinase-3) in glioma models; ANP32A and SMAR-CA4 in B-cell lymphoma models, as well as some members of p53- and TGFβ-associated pathways regulating apoptosis [61]. Moreover, miR-21 seems to be associated with the development of tumor resistance to chemotherapy, and suppression of microRNA expression led to tumor regression. Therefore, miR-21 is a promising target for selective inhibition in anti-tumor therapy.

At the same time, there are microRNAs with antitumor activity. The most important of them is miR-34a, the decreased expression of which has been detected in many tumors, including glioblastoma, and the level of miR-34a expression can serve as a prognostic sign [62,63]. miR-34a is closely related to the activity of p53-protein, and both p53 has an activating effect on microRNAs and regulates the expression of several p53-dependent genes, thus forming positive feedback loops [64]. Moreover, the targets of miR-34 can be molecules responsible for tumor proliferation and invasion, as well as epithelial-mesenchymal transition (EMT) (vimentin and E-cadherin in bladder and colon cancer models) [62]. miR-34 induction in Hep3B and HuH7-induced liver tumor models in mice resulted in tumor growth retardation and tumor regression [65], and induction of miR-34 expression in adriamycin-resistant MCF-7 cells restored their sensitivity to chemotherapy [66]. That makes the exogenous miR-34a a promising antitumor entity.

As carriers, we have chosen polycationic phosphorus and carbosilane dendrimers that we had already proven to be efficient carriers of therapeutic nucleic acids into both tumor [38,67] and immunocompetent cells [25,31,33,41,67].

As models for assessing anti-glioblastoma activity of dendrimer-microRNA complexes, we have chosen glioblastoma cell cultures and glioblastoma stem-like cells. These cell lines are cultured as a suspension in DMEM/F12 serum-free medium. This minimizes the risk of changes in complex behavior due to the formation of a protein corona. Furthermore, they form 3D structures called neurospheres. The existence of such structures makes it possible to evaluate the effects of drugs in a more complex three-dimensional system, which is an undoubted advantage when extrapolating the data obtained on cellular models to real objects.

Of particular interest is the possibility to work with tumor stem cells, a subpopulation of tumor cells capable of self-sustaining and associated with tumor formation, progression and recurrence [3,4,5,6,7]. It is also currently suggested that tumor stem cells may play a key role in the development of glioblastoma resistance to chemotherapy [5,6,7,8,9,10]; and different model tumor lines may have different sensitivity to drugs [11,68]. Cell lines under study differ in their molecular and biological characteristics, belonging to different glioblastoma subtypes.

We used the human glioblastoma cell line U87 and induced human pluripotent stem cells (iPSCs) as comparison groups. The U87 line cells are interesting because they are usually cultured in a medium containing fetal bovine serum and represent a classical adherent culture, but when using serum-free DMEM/F12 medium they behave as a suspension culture forming neurospheres [69], which allows one to comapre cultures with high and low content of cells with pluripotent potential [70]. iPSCs represent a relatively convenient object for comparing pluripotent cells of tumor and non-tumor nature; these cells are also cultured under serum-free conditions, but they represent an adherent culture.

Evaluation of the biological effects of dendrimers and their complexes with microRNAs miR-34a and amiR-21 included a number of key parameters traditionally investigated in preclinical trials of new therapeutic substances. We evaluated how dendrimers/dendriplexes affect tumor cell viability and apoptosis induction parameters compared to temozolomide, a standard glioblastoma care drug. At the same time, in our opinion, it is important to study the parameters characterizing interactions between the tumor and its microenvironment, in particular, with immunocompetent cells. Based on the literature data, we chose several parameters: expression of surface markers (PD-L1, TIM3, CD47) and IL-10 secretion [38]. An important aspect related to tumor immune escape is the secretion of factors with immunosuppressive activity: IL-10, TGF beta, IDO. [8,54]. Increased levels of IL-10 are known in patients with glioblastoma [71], what makes this parameter important for evaluation in this work.

Our proof-of-concept study shows that dendrimer-based nanoformulations affect the viability of GSCs, glioblastoma cells and iPSCs in a dose-dependent manner. Although the effects of formulations containing antitumor oligonucleotides seem less evident in an oxidative potential-based assay (MTT), apoptosis tests evidence that the ratio of cells at the early apoptosis stage significantly increase upon the incubation with miR-34a and anti-miR-21-containing dendriplexes. This has useful implications for cancer therapy, as controlled induction of programmable cell death is preferential over acute non-specific cytotoxicity, frequently observed for chemodrugs.

Furthermore, we have performed a study of dendrimer effects on the expression of surface markers in GSCs that are responsible for their interaction with the tumor niche. At the moment, we cannot definitely assign the dynamics of surface markers expression to the appropriateness of distinct formulations for glioblastoma therapy, however, we believe that these data can provide useful information for the design of antitumor nanoformulations in future, when compared with further findings.

## 5. Conclusions

In the present study, we have performed a preclinical characterization of the biological effects of dendrimer-based nanoformulations in a panel of glioblastoma and glioma cells, glioblastoma stem-like cells and induced pluripotent stem cells. Dendrimers showed their efficacy as independent antitumor agents. They were observed to be effective carriers of anti-glioblastoma microRNA and microRNA inhibitors. Importantly, upon treatment, we have observed certain changes in the expression of surface markers that characterize the interaction of tumor cells with the immune microenvironment. Although the role of these changes in antitumor activity in vitro and in vivo is not quite clear yet, we believe that affecting tumor-microenvironment interactions is a crucial parameter for clinical use worth further investigation in view of the development of dendrimer-based therapeutic constructions.

## Data Availability

Data supporting the conclusions of this article will be made available by the authors upon reasonable request.

## Supplementary Materials

The following supporting information can be downloaded at: https://www.mdpi.com/article/10.3390/pharmaceutics15030968/s1, Primary data for Figure 2, Figure 3, Figure 4, Figure 5, Figure 6, Figure 7, Figure 8, Figure 9, Figure 10, Figure 11, Figure 12, Figure 13 and Figure 14.
