# Supplementary material for: Effects of Dendrimer-microRNA Nanoformulations against Glioblastoma Stem Cells
Source: Pharmaceutics. 2023 Mar 17;15(3):968. doi: 10.3390/pharmaceutics15030968 (PMC10056969; doi:10.3390/pharmaceutics15030968)
Supplement: Supplementary file 1 [file pharmaceutics-15-00968-s001.zip › pharmaceutics-2201858-supplementary.pdf]

Supporting Information:

Effects of Dendrimer-microRNA Nanoformulations Against Glioblastoma Stem Cells

Nadezhda Knauer, Mariya Meschaninova, Sajjad Muhammad, Daniel Hänggi, Jean-Pierre Majoral, Ulf Dietrich Kahlert, Vladimir Kozlov, Evgeny K. Apartsin

Primary data for the Figure 2: Assessment of effects of cationic phosphorus (AE2G3) and carbosilane (BDEF33) dendrimers on the viability of glioblastoma tumor cell lines and iPS cells (iPSCs) after 72 h co-culture compared with the standard chemotherapy drug temozolomide (TMZ), data are presented as percent of value for the NTC.

BTSC233						
	AE2G3		BDEF33		TMZ	
	Mean	S.D.	Mean	S.D.	Mean	S.D.
NTC	95.2	5.1	100.0	9.6	100.0	17.6
0.1 µM	90.0	18.8	91.4	4.4	58.8	11.6
1 µM	87.4	15.6	93.7	9.4	94.7	31.2
10 µM	1.8	1.8	1.3	2.1	118.7	20.6
100 µM	48.9	17.6	1.8	1.4	86.1	28.4

JHH520						
	AE2G3		BDEF33		TMZ	
	Mean	S.D.	Mean	S.D.	Mean	S.D.
NTC	100.0	24.3	100.0	12.6	100.0	48.7
0.1 µM	80.7	2.8	104.3	13.0	100.9	0.0
1 µM	69.4	9.4	75.0	8.9	121.5	41.7
10 µM	1.9	1.5	0.0	0.1	81.1	25.1
100 µM	35.1	11.5	4.2	2.9	133.4	67.8

NCH644						
	AE2G3		BDEF33		TMZ	
	Mean	S.D.	Mean	S.D.	Mean	S.D.
NTC	97.1	5.0	100.0	11.6	100.0	13.7
0.1 µM	63.7	20.1	86.7	10.0	104.0	17.3
1 µM	33.1	2.3	83.9	5.0	98.2	8.5
10 µM	12.2	3.9	1.3	1.1	101.4	7.6
100 µM	87.2	17.1	2.3	1.6	83.8	16.1

SF188						
	AE2G3		BDEF33		TMZ	

	Mean	S.D.	Mean	S.D.	Mean	S.D.
NTC	121.1	11.7	100.0	7.6	87.0	8.3
0.1 µM	89.3	7.3	116.5	16.5	70.8	4.5
1 µM	30.2	14.5	98.1	15.8	82.5	13.4
10 µM	0.0	0.0	0.5	0.7	162.9	33.9
100 µM	53.8	10.6	2.4	1.8	51.5	4.5

GBM1						
	AE2G3		BDEF33		TMZ	
	Mean	S.D.	Mean	S.D.	Mean	S.D.
NTC	84.6	16.1	100.0	7.4	100.0	10.0
0.1 µM	103.5	9.1	79.2	5.5	93.9	15.2
1 µM	89.0	11.2	88.7	15.2	123.4	12.9
10 µM	6.4	7.2	93.1	7.5	115.4	2.7
100 µM	8.9	4.7	2.8	2.5	117.5	3.5

U87						
	AE2G3		BDEF33		TMZ	
	Mean	S.D.	Mean	S.D.	Mean	S.D.
NTC	100.0	26.2	100.0	14.3	97.1	5.4
0.1 µM	119.9	9.3	107.5	10.5	105.8	16.8
1 µM	90.6	7.4	59.1	25.9	86.9	13.0
10 µM	45.8	18.7	0.1	0.2	66.1	9.7
100 µM	14.0	1.5	1.3	2.1	75.1	8.9

iPS						
	AE2G3		BDEF33		TMZ	
	Mean	S.D.	Mean	S.D.	Mean	S.D.
NTC	100.0	27.0	100.0	27.0	110.3	11.2
0.1 µM	122.0	9.9	115.4	18.9	89.5	19.9
1 µM	95.9	8.1	111.6	12.7	66.6	16.9
10 µM	4.8	1.3	2.9	1.5	78.2	21.7
100 µM	11.9	10.3	1.4	1.7	96.7	0.0

Primary data for the Figure 1. Assessment of apoptosis induction parameters of glioblastoma tumor cell lines and iPSCs after 72 h of co-culture with free dendrimers compared to standard chemodrug temozolomide (TMZ); concentration of substances tested is 10 μM.

BTSC233						
	Live cells		Early apoptotic		Late apoptotic/Dead	
	Mean	S.D.	Mean	S.D.	Mean	S.D.
NTC	55.6	3.1	16.0	2.1	27.7	4.0
AE2G3	27.0	5.1	5.6	1.2	65.9	5.5
BDEF33	41.0	8.5	6.9	0.6	50.9	8.3
TMZ	64.3	1.7	12.2	3.6	22.4	1.3

JHH520						
	Live cells		Early apoptotic		Late apoptotic/Dead	
	Mean	S.D.	Mean	S.D.	Mean	S.D.
NTC	72.1	1.6	16.5	6.0	10.8	6.6
AE2G3	51.7	1.2	24.0	2.7	24.2	2.3
BDEF33	50.8	3.3	20.7	0.8	28.4	2.5
TMZ	66.4	4.4	22.2	3.9	11.4	0.6

NCH644						
	Live cells		Early apoptotic		Late apoptotic/Dead	
	Mean	S.D.	Mean	S.D.	Mean	S.D.
NTC	86.4	4.3	8.4	2.4	5.1	1.9
AE2G3	42.7	5.2	27.0	1.7	30.1	3.7
BDEF33	45.8	6.0	23.0	2.1	31.0	3.9
TMZ	86.9	1.9	8.0	1.8	5.0	0.3

U87						
	Live cells		Early apoptotic		Late apoptotic/Dead	
	Mean	S.D.	Mean	S.D.	Mean	S.D.
NTC	73.5	12.6	15.7	7.5	10.5	5.4
AE2G3	48.7	2.6	29.9	11.7	21.3	12.0
BDEF33	69.4	4.0	10.6	0.2	19.9	3.8
TMZ	66.6	4.8	24.5	4.4	8.8	0.5

iPS						
	Live cells		Early apoptotic		Late apoptotic/Dead	
	Mean	S.D.	Mean	S.D.	Mean	S.D.

NTC	72.7	2.5	15.0	2.4	12.2	0.1
AE2G3	76.8	0.2	7.3	1.6	14.4	1.6
BDEF33	63.0	8.1	26.5	4.8	10.5	6.7
TMZ	55.0	0.7	29.9	0.8	12.4	4.6

Primary data for the Figure 2. Assessment of PD-L1 marker expression on the surface of glioblastoma tumor cell lines and iPSCs after 72 h of co-culture with free dendrimers compared to standard chemodrug temozolomide (TMZ); concentration of the substances tested is 3 μM.

	GBM1		U87		NCH644		iPS	
	Mean	S.D.	Mean	S.D.	Mean	S.D.	Mean	S.D.
NTC	14.1	0.4	0.9	0.1	2.5	0.9	3.1	1.0
AE2G3	1.0	0.8	0.4	0.1	71.6	28.4	22.9	2.6
BDEF33	1.7	1.4	0.6	0.0	5.1	2.3	28.9	10.5
TMZ	4.6	1.6	0.3	0.1	1.4	0.2	2.3	0.7

Primary data for the *Figure 3*. Evaluation of TIM3, CD47 expression on the surface of NCH644 tumor cell lines after 72 h of co-culture with free dendrimers compared to standard chemodrug temozolomide (TMZ); concentration substances tested is 3 μM.

	TIM3		CD47	
	Mean	S.D.	Mean	S.D.
NTC	1.2	0.1	25.2	3.8
AE2G3	84.1	4.2	43.8	11.2
BDEF33	1.2	0.9	29.4	4.6
TMZ	0.8	0.1	21.8	5.3

Primary data for the *Figure 4*. Assessment of IL-10 cytokine secretion in NCH644 tumor cell cultures after 72 h of co-culture with free dendrimers compared to standard chemodrug temozolomide (TMZ)

	Mean	S.D.
NTC	100.0	0.0
AE2G3	82.1	18.0
BDEF33	85.2	14.3
TMZ	101.4	30.9

Primary data for the *Figure 5*. Assessment of the efficiency of internalization of dendrimer complexes with fluorescently labeled microRNA (miR-155-FAM) into tumor cells and iPS cells compared to Lipofectamine 3000 (Lipo) (4 h).

BTSC233		
	Mean	S.D.
NTC	0.00	0.00
miR	0.84	0.06
AE2G3	0.04	0.06
AE2G3/miR	10.47	0.44
BDEF33	0.00	0.00
BDEF33/miR	2.14	0.59
Lipo	0.00	0.00
Lipo/miR	2.49	1.80

GBM1		
	Mean	S.D.
NTC	0.15	0.13
miR	21.25	5.80
AE2G3	0.15	0.06
AE2G3/miR	69.76	1.59
BDEF33	0.95	0.56
BDEF33/miR	1.47	0.69
Lipo	0.84	0.41
Lipo/miR	6.41	0.52

iPS		
	Mean	S.D.
NTC	0.00	0.00
miR	2.91	0.13
AE2G3	1.20	0.24
AE2G3/miR	4.74	1.44
BDEF33	1.10	0.64
BDEF33/miR	24.73	4.84
Lipo	0.47	0.15
Lipo/miR	12.83	0.36

JHH520		
	Mean	S.D.
NTC	0.15	0.20
miR	2.10	0.82
AE2G3	0.05	0.08
AE2G3/miR	39.06	5.47
BDEF33	0.08	0.05
BDEF33/miR	5.34	1.19
Lipo	0.09	0.07
Lipo/miR	2.20	0.44

U87		
	Mean	S.D.
NTC	0.09	0.04
miR	51.67	5.91
AE2G3	0.73	0.18
AE2G3/miR	50.41	4.70
BDEF33	0.60	0.33
BDEF33/miR	1.04	0.06
Lipo	1.19	1.60
Lipo/miR	40.22	6.31

Primary data for the *Figure 6*. Evaluation of the effects on the viability of GBM1 tumor cell line after 72 h co-culture with dendriplexes.

	AE2G3/miR34		AE2G3/amiR21		BDEF33/miR34		BDEF33/amiR21	
	Mean	S.D.	Mean	S.D.	Mean	S.D.	Mean	S.D.
NTC	79.0	14.1	84.6	16.1	100.0	10.9	100.0	10.9
mock	104.5	12.8	104.5	12.8	126.9	12.8	126.9	12.8
miR	114.6	15.1	116.7	39.6	114.6	15.1	116.7	39.6
25	91.2	24.1	106.7	16.6	86.0	15.6	87.1	18.8
50	108.9	22.0	92.1	15.3	101.4	23.7	76.3	18.3
100	127.3	26.7	77.9	16.2	104.6	13.6	64.6	13.3
150	108.2	7.8	77.7	10.3	80.5	15.8	53.2	11.2

Primary data for the *Figure 7*. Evaluation of apoptosis induction parameters of GBM1 tumor cells after 72 h of co-culture with dendriplexes.

	Live cells		Early apoptotic		Late apoptotic/Dead	
	Mean	S.D.	Mean	S.D.	Mean	S.D.
NTC	91.48	1.73	7.36	1.80	1.11	0.12
miR-34	94.33	1.93	4.93	1.64	0.73	0.29
amiR-21	92.11	2.03	7.39	1.92	0.49	0.20
AE2G3	90.02	0.72	9.04	0.48	0.93	0.25
AE2G3/miR-34	90.93	0.61	8.31	0.62	0.76	0.06
AE2G3/amiR-21	90.02	0.87	9.14	0.98	0.85	0.22
BDEF33	85.71	0.80	13.03	0.37	1.23	0.51
BDEF33/miR-34	72.86	2.48	26.20	2.38	0.94	0.16
BDEF33/amiR-21	73.00	0.96	25.54	0.83	1.45	0.39

Primary data for the Figure 8. Evaluation of the effects on the viability of JHH520 tumor cell line after 72 h co-culture with dendriplexes

	AE2G3/miR34		AE2G3/amiR21		BDEF33/miR34		BDEF33/amiR21	
	Mean	S.D.	Mean	S.D.	Mean	S.D.	Mean	S.D.
NTC	100.0	18.0	100.0	18.0	100.0	16.4	100.0	16.4
mock	113.9	6.0	113.9	6.0	87.4	10.9	87.4	10.9
miR	88.9	6.8	95.9	4.0	88.9	6.8	95.9	4.0
25	112.4	10.6	99.4	7.0	85.1	15.1	86.3	23.3
50	100.7	7.2	96.1	4.4	95.5	12.4	82.1	9.6
100	109.1	9.1	104.1	11.8	87.4	10.8	81.2	4.8
150	95.4	6.9	96.8	4.0	95.0	18.8	93.6	8.6

Primary data for the *Figure 9*. Evaluation of the effects on the viability of NCH644 tumor cell line after 72 h co-culture with dendriplexes

	AE2G3/miR34		AE2G3/amiR21		BDEF33/miR34		BDEF33/amiR21	
	Mean	S.D.	Mean	S.D.	Mean	S.D.	Mean	S.D.
NTC	100.0	21.6	100.0	21.6	100.0	15.8	100.0	15.8
mock	85.8	6.3	85.8	6.3	62.9	11.0	62.9	11.0
miR	100.8	13.6	112.2	14.0	100.8	13.6	112.2	14.0
25	104.7	6.1	89.9	7.3	93.8	12.4	81.0	4.4
50	91.9	11.7	102.1	12.4	90.3	5.3	81.7	4.7
100	94.2	7.0	90.3	9.1	79.0	9.0	82.2	10.9
150	85.7	11.3	74.7	11.0	82.4	7.6	73.8	7.9

Primary data for the *Figure 10*. Evaluation of the effects on the viability of U87 tumor cell line after 72 h co-culture with dendriplexes

	AE2G3/miR34		AE2G3/amiR21		BDEF33/miR34		BDEF33/amiR21	
	Mean	S.D.	Mean	S.D.	Mean	S.D.	Mean	S.D.
NTC	99.1	10.9	99.1	10.9	116.1	19.8	116.1	19.8
mock	86.5	23.4	86.5	23.4	162.8	15.7	162.8	15.7
miR	82.2	18.0	90.7	14.7	82.2	18.0	90.7	14.7
25	80.2	16.5	70.3	16.4	79.1	26.2	126.1	15.3
50	65.1	19.4	72.2	14.8	126.5	8.5	120.9	11.6
100	65.1	16.9	67.4	10.4	116.0	11.0	124.7	41.2
150	51.5	10.2	62.5	14.9	91.4	21.5	90.3	26.4

Primary data for the *Figure 11*. Evaluation of apoptosis induction parameters of U87 tumor cells after 72 h of co-culture with dendriplexes

	Live cells		Early apoptotic		Late apoptotic/Dead	
	Mean	S.D.	Mean	S.D.	Mean	S.D.
NTC	84.97	3.17	7.56	1.41	6.77	3.91
miR-34	90.99	1.80	6.54	1.07	2.32	0.67
amiR-21	88.17	3.73	8.46	2.57	3.08	1.06
AE2G3	69.61	5.49	26.16	5.02	4.20	0.69
AE2G3/miR-34	72.73	3.15	21.56	2.38	5.67	2.20
AE2G3/amiR-21	75.89	0.40	19.08	2.73	4.97	3.04
BDEF33	78.01	2.11	18.21	1.78	3.75	0.86
BDEF33/miR-34	69.79	0.90	22.51	0.88	7.68	0.12
BDEF33/amiR-21	67.23	2.26	23.06	3.51	9.66	1.80

Primary data for the *Figure 12*. Evaluation of PD-L1 marker expression on the surface of GBM1 and U87 tumor cells after 72 h of co-culture with dendriplexes.

	GBM1		U87	
	Mean	S.D.	Mean	S.D.
NTC	28.45	1.95	1.50	0.17
miR-34	21.17	1.29	3.15	0.32
amiR-21	7.90	1.62	1.36	0.31
AE2G3	7.71	2.32	1.07	0.17
AE2G3/miR-34	8.00	0.29	1.01	0.15
AE2G3/amiR-21	3.86	1.56	0.86	0.13
BDEF33	24.65	3.43	1.18	0.07
BDEF33/miR-34	3.05	1.91	0.55	0.06
BDEF33/amiR-21	1.82	0.23	0.35	0.04
